# Effect of sulfadoxine-pyrimethamine doses for prevention of malaria during pregnancy in hypoendemic area in Tanzania

**DOI:** 10.1186/s12936-020-03234-4

**Published:** 2020-04-19

**Authors:** Wigilya P. Mikomangwa, Omary Minzi, Ritah Mutagonda, Vito Baraka, Eulambius M. Mlugu, Eleni Aklillu, Appolinary A. R. Kamuhabwa

**Affiliations:** 1 Clinical Pharmacy and Pharmacology Department, Muhimbili University of Health and Allied Sciences, Dar es Salaam, United Republic of Tanzania; 2 National Institute for Medical Research, Tanga Centre, Tanga, United Republic of Tanzania; 3 Pharmaceutics and Pharmacy Practice Department, Muhimbili University of Health and Allied Sciences, Dar es Salaam, United Republic of Tanzania; 4 Division of Clinical Pharmacology, Department of Laboratory Medicine, Karolinska Institutet, Karolinska University Hospital-Huddinge C1:68, 141 86 Stockholm, Sweden

**Keywords:** Malaria, Pregnancy, Intermittent-preventive treatment, Sulfadoxine-pyrimethamine, Anaemia, Tanzania

## Abstract

**Background:**

Malaria in pregnancy increases the risk of deleterious maternal and birth outcomes. The use of ≥ 3 doses of sulfadoxine-pyrimethamine (SP) for intermittent preventive treatment of malaria (IPTp-SP) is recommended for preventing the consequences of malaria during pregnancy. This study assessed the effect of IPTp-SP for prevention of malaria during pregnancy in low transmission settings.

**Methods:**

A cross-sectional study that involved consecutively selected 1161 pregnant women was conducted at Mwananyamala regional referral hospital in Dar es Salaam. Assessment of the uptake of IPTp-SP was done by extracting information from antenatal clinic cards. Maternal venous blood, cord blood, placental blood and placental biopsy were collected for assessment of anaemia and malaria. High performance liquid chromatography with ultraviolet detection (HPLC-UV) was used to detect and quantify sulfadoxine (SDX). Dried blood spots (DBS) of placental blood were collected for determination of sub-microscopic malaria using polymerase chain reaction (PCR).

**Results:**

In total, 397 (34.2%) pregnant women reported to have used sub-optimal doses (≤ 2) while 764 (65.8%) used optimal doses (≥ 3) of IPTp-SP at the time of delivery. The prevalence of placental malaria as determined by histology was 3.6%. Submicroscopic placental malaria was detected in 1.4% of the study participants. Women with peripheral malaria had six times risk of maternal anaemia than those who were malaria negative (aOR, 5.83; 95% CI 1.10–30.92; p = 0.04). The geometric mean plasma SDX concentration was 10.76 ± 2.51 μg/mL. Sub-optimal IPTp-SP dose was not associated with placental malaria, premature delivery and fetal anaemia. The use of ≤ 2 doses of IPTp-SP increased the risk of maternal anaemia by 1.36-fold compared to ≥ 3 doses (aOR, 1.36; 95% CI 1.04–1.79; p = 0.02).

**Conclusion:**

The use of < 2 doses of IPTp-SP increased the risk of maternal anaemia. However, sub-optimal doses (≤ 2 doses) were not associated with increased the risk of malaria parasitaemia, fetal anaemia and preterm delivery among pregnant women in low malaria transmission setting. The use of optimal doses (≥ 3 doses) of IPTp-SP and complementary interventions should continue even in areas with low malaria transmission.

## Background

Despite the efforts to control and eliminate malaria worldwide, malaria in pregnancy (MiP) has remained a significant contributor of maternal, neonate and infant morbidity and mortality. The recent World Health Organization (WHO) malaria report indicated that 219 million malaria cases and 435,000 deaths were reported worldwide, of which 80% were from sub-Saharan Africa and India [1]. Following the implementation of integrated strategies to curb malaria globally, some regions have attained low level of malaria transmission [1]. In Tanzania, the prevalence of malaria has declined for more than 50% in 10 years (7.3%, in 2017 compared to 18% in 2007) [2, 3]. The implemented strategies include the use of insecticide-treated nets (ITNs), indoor residual spray and larval source reduction using biolarvicides [4]. The other major strategies include intermittent preventive treatment of malaria in pregnant women using sulfadoxine-pyrimethamine (IPTp-SP) and prompt malaria diagnosis and treatment with effective anti-malarial drugs [5].

Sulfadoxine (1500 mg)/Pyrimethamine (75 mg) is given to pregnant women as a single therapeutic dose on direct observed therapy (DOT) during visits to the antenatal clinics (ANC). The drug is readily absorbed (bioavailability > 90%) and reaches peak plasma concentration of about 183 μg/mL (sulfadoxine) and 0.55 ng/mL (pyrimethamine) 2–8 h after oral administration [6]. Sulfadoxine (SDX) is eliminated through glomerular filtration and 70% of it undergoes tubular reabsorption which contributes to its long elimination half-life [6–8]. SDX can stay in the plasma for up to 9 weeks, while pyrimethamine is fast cleared and up to 30% excreted through the urine [7–9]. For SP to be safe and well tolerated, the doses of IPTp-SP should be administered from the earliest second trimester (14 weeks of gestation) to delivery, with each dose given at one-month interval [10–12].

The beneficial effect of IPTp-SP is believed to be due to suppression rather than complete clearance of parasites in the peripheral and placenta [10, 11]. The uptake of IPTp-SP during the course of pregnancy prevents deleterious maternal and fetal outcomes associated with malaria in pregnancy (MiP) [13–15]. The widespread SP resistance due to mutations in the parasite’s *dihydrofolate reductase* (*Pfdhfr*) and *dihydropteroate synthase* (*Pfdhps*) genes, rendered the previous recommended 2-doses ineffective in averting adverse birth and maternal outcomes due to MiP [16–19]. Instead, the uptake of at least three doses of IPTp-SP (≥ 3 doses) during pregnancy was considered optimum [19]. The IPTp-SP ≥ 3 doses are associated with reduced odds of adverse birth outcomes such as low birth weight (LBW) and maternal anaemia in areas with moderate to high malaria transmission [20–22]. However, such beneficial effects of IPTp-SP uptake in low malaria transmission (hypoendemic) have not been fully explored.

The impact of IPTp-SP on birth outcomes among pregnant women living in moderate to high malaria transmission areas have been widely studied. There still exists the question on the benefit of using IPTp-SP in areas with substantial reduction of malaria transmission. To cover this knowledge gap, the study describes the influence of optimal and sub-optimal uptake of IPTp-SP doses on MiP as well as on the adverse birth outcomes among pregnant women living in low malaria transmission in Dar es Salaam, Tanzania.

## Methods

### Study design, study population and study area

This was a cross-sectional study conducted between April and November, 2018 and involved 1161 pregnant women with at least 18 years of age, admitted in the delivery unit at Mwananyamala regional referral hospital in Kinondoni Municipality, in Tanzania. Pregnant women in delivery wards were recruited consecutively. Those who had complicated pregnancy with high risk of haemorrhage, preeclampsia, eclampsia, HIV positive, with incomplete ANC cards (not included either IPTp-SP use, mebendazole use, FEFO use or gravidity, delivered twins, delivered by caesarian section, resided in Dar es Salaam less than six months and used co-trimoxazole were excluded from the study. Dar es Salaam region is considered a low malaria transmission area with malaria prevalence of 1.1% among children below 5 years of age [3]. As per the WHO, low malaria transmission is when the prevalence of malaria in below 10% among children aged 2–9 years [5]. The region generally experiences tropical climatic conditions, typified by hot and humid weather throughout much of the year with an average temperature of 29 °C. Annual rainfall is approximately 1100 mm (lowest 800 mm and highest 1,300 mm), and in a normal year there are two rainy seasons: the long rains from March/April to May and the short rains from October to November/December [20]. Therefore, the study was conducted to cover both rain and dry season which could have influenced malaria transmission.

### Data collection

Validated case report form (CRF) was used to collect information on socio-demographic characteristics such as place of residence, age, gender, marital status and education level; obstetric characteristics; the use of anaemia preventive measures example FEFO (Ferrous, 60 mg/folic acid, 400 µg) and mebendazole, 500 mg; malaria preventive measures including IPTp-SP, mosquito repellents or spray and ITN; the number of IPTp-SP doses used as documented on ANC card; history on the previous malaria infection and anti-malarial used; and, birth outcomes. Gestation age was determined by last normal menstrual period and documented.

### Blood sample collection and haemoglobin determination

Three EDTA tubes were used to collect maternal blood, cord blood (fetal blood) and placental blood. The EDTA tubes were inverted ten times (to enable mixing of whole blood with EDTA) then transferred to the laboratory at Mwananyamala Regional Referral Hospital for malaria and haemoglobin examination. Maternal and fetal Hb levels were determined using HemoCue^®^ Hb 201 + HemoCue AB, Angelholm, Sweden. The WHO cut off points on haemoglobin were used to characterize maternal and fetal haemoglobin status as normal or anaemia [21]. Pregnant women and fetus were considered anaemic at Hb < 11.0 g/dL and Hb < 12.5 g/dL, respectively [21, 22].

### Assessment of maternal peripheral malaria

Laboratory analysis of blood samples Maternal and fetal Hb levels were determined using HemoCue^®^ Hb 201 + HemoCue AB, Angelholm, Sweden. About 5 μL of blood was used for testing malaria infection using malaria Rapid diagnostic test, (RDT), SD BIOLINE Malaria Ag P.f/pan, Standard Diagnostics, INC. Microscopy was used to confirm the results of RDT. Thick and thin blood smears were stained with 2% Giemsa. Microscopic examination was performed by two competent laboratory technologists, any discordant in results was resolved by the third reader. A blood smear was considered negative when the examination of 100 high power fields did not reveal asexual parasites.

### Assessment of placental malaria by histology

After delivery, placenta was sliced using a disposable lancet halfway between the edge of placenta and insertion of the cord. The placental biopsy with approximately 2 cm^3^ was placed in a container with 10% neutral buffered formalin and stored at room temperature for transfer and processing at Muhimbili University of Health and Allied Sciences (MUHAS) pathology laboratory. Paraffin-embedded placental specimens were sectioned, stained with haematoxylin and eosin (H&E) and Giemsa. Malaria parasites and pigments were examined under light microscopy. Malaria parasites were identified by their presence in the erythrocytes in intervillous space. On the other hand, malaria pigments were identified by their presence in the erythrocytes and monocytes in intervillous space and pigment in fibrin [23]. Two experienced laboratory scientists assessed placental malaria. The third laboratory scientist was involved to read the slides that had discordant readings between the two readers. Placental malaria was recorded as infected RBCs, haemozoin and infected RBCs with haemozoin pigment.

### Assessment of placental malaria by PCR

Two drops of placental blood were spotted on a filter paper (Whatman^®^3MM, Maidstone, UK), air dried overnight and preserved in plastic bags for PCR analysis. After histological analysis of placental biopsy for placental malaria, 286 DBS samples with negative placental malaria were randomly selected to include participants who used ≤ 2 and ≥ 3 IPTp-SP doses. The 286 DBS samples were used to determine submicroscopic malaria infection by PCR. The DBS were transported to the laboratory at the National Institute for Medical Research, Tanga Centre for detection of submicroscopic malaria infection. The DNA was extracted from DBS using the QIAamp DNA Minikit (Qiagen, Hilden, Germany) following the manufacturer’s protocol. DNA was eluted in 150 μl of buffer and samples were stored at − 20 °C freezer until time of use. The *Plasmodium* species were identified using nested PCR according to the Snounou et al. protocol [24].

### Determination of plasma sulfadoxine

Aliquots of maternal venous blood were collected and centrifuged at 2000 g for 10 min [25]. Plasma was stored in 2ml cryotubes and stored at − 20 ℃ at MUHAS, Sida-bioanalytical laboratory for analysis. High Performance Liquid Chromatography with Ultraviolet detection (HPLC-UV) technique was used as described by Virendra et al. [25] with minor modifications. The extraction process used a mixture of diethyl ether and ethyl acetate at the ratio of 2:1 and a reversed-phase column (ReproSil-Pur Basic C18, 5 μ, 250 × 4.6 mm) was used. Sulfamethoxazole was used as internal standard, and the low limit of quantification (LLoQ) and low limit of detection (LLoD) was 3 μg/ml and 0.5 μg/ml, respectively. Plasma samples were analysed together with quality controls and calibration standards. Detection of SDX was used as a proxy for the use of SP for IPTp. SDX can be detected in plasma up to 63 days from the last dose. On the other hand, pyrimethamine has short half-life of 3 to 5 days, and can be detected up to 42 days after oral intake [6, 26]. Studies have reported the median concentration of SDX among pregnant women 7 days and 42 days after the last dose of SP to be approximately 75 μg/ml and 2 μg/ml, respectively [25, 26].

### Data analysis

The primary outcome of the study was placental malaria and the secondary outcomes were adverse birth outcomes including maternal anaemia, fetal anaemia and premature delivery. Plasma SDX concentrations were log-transformed and the geometric means with standard deviation were presented. Chi square and Fischer exact tests were used to compare grouped data (such as gestation age, anaemia status, age groups, gravidity, marital status and IPTp-SP dose groups). Spearman’s correlation was used to establish the relationship between continuous variables such as maternal hemoglobin concentration and SDX concentration. Variables that had p-value ≤ 0.2 were subjected to multivariate analysis to establish their influence on the relationship between IPTp-SP doses and outcomes of interest. Multivariable logistic regression was performed to determine the effect of IPTp-SP doses on placental malaria, maternal anaemia, fetal anaemia and preterm delivery. Significance level was set at 0.05 and the confidence level at 95%. Exposure variables that had p-value < 0.05 were considered significant predictor of the outcome variables. The IPTp-SP doses were grouped as sub-optimal and optimal doses when participants used ≤ 2 doses and ≥ 3 doses, respectively. Analysis was conducted using a Statistical Package for Social Sciences (SPSS) program version 23.0.

## Results

### Socio-demographic and obstetric characteristics of study participants

The total of 1161 pregnant women participated in the study with median (IQR) age of 25 (18–44) years old. Most of them were married (73.7%) with primary education level (60.9%) and attended ANC visits at least 4 times (66.9%) during their recent pregnancies. They reported to have used various malaria preventive measures, such as mosquito sprays/repellents (57.9%) and ITN (98.1%), and anaemia preventive strategies including mebendazole (96.6%) and FEFO (97.9%) (Table 1). Twenty-eight (2.4%) women reported to have contracted malaria at least once during their recent pregnancies; 19 used artemether-lumefantrine, 3 artesunate injection, 1 quinine tablets, 1 dihydroartemisinin-piperaquine (DP) and 4 could not recall the names of the anti-malarial drugs they used. Fifteen (1.3%) pregnant women had fever with no confirmed no malaria.

**Table 1:** Socio-demographic and obstetric characteristics of study participants

Characteristics	n	%
Age groups (years)		
18–24	526	45.3
25–29	304	26.2
30–34	208	17.9
> 34	123	10.6
Body temperature (°C)		
36.5–37.5	1146	98.7
> 37.5	15	1.30
Marital status		
Married	856	73.7
Unmarried	305	26.3
Education level		
No formal education	29	2.5
Primary education	707	60.9
Secondary education	397	34.2
Tertiary education	28	2.4
Attendance to ANC		
< 4	384	33.1
≥ 4	777	66.9
FEFO use		
Yes	1137	97.9
No	24	2.1
Mebendazole use		
Yes	1121	96.6
No	40	3.4
Gravidity		
Primigravida	446	38.4
Secundigravida	328	28.3
Multigravida	387	33.3
ITN use		
Yes	1139	98.1
No	22	1.9
Mosquito spray/repellant		
Yes	672	57.9
No	489	42.1
Gestation age (weeks)		
≤ 36	90	7.8
≥ 37	1071	92.2
Sex of newborn		
Male	591	50.9
Female	570	49.1

*ANC* antenatal clinic*, FEFO* ferrous/folic acid, *ITN* insecticide treated nets

At delivery, half of the women gave birth to male or female babies at mean (± SD) gestation age of 38.8 (± 1.6) weeks with birth weight of 3.1 (± 0.46) Kg. The proportions of primigravida, secundigravida and multigravida among the study participants were 38.4%, 28.3% and 33.3%, respectively (Table 1).

### The uptake of optimal IPTp-SP doses

**Fig. 1:**
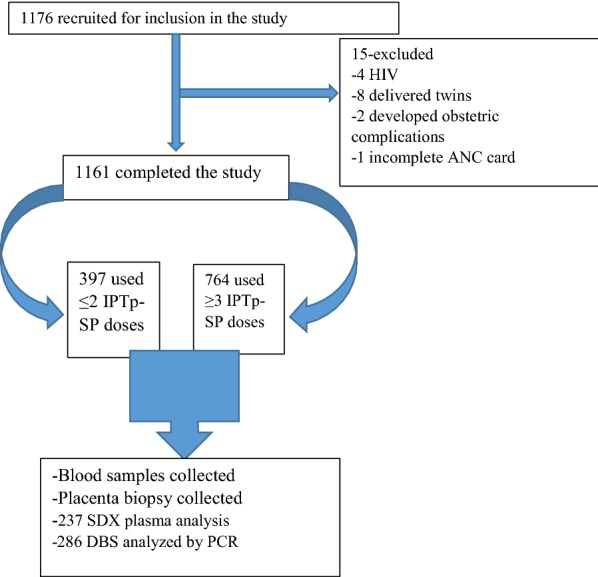
Study flow chart

The total of 397 (34.2%) women used ≤ 2 doses while 764 (65.8%) used ≥ 3 doses of IPTp-SP (Fig. 1). Most of the participants’ characteristics such as participants age, gravidity and education level were not associated with the uptake of IPTp-SP (p > 0.05). On univariate analysis the uptake of IPTp-SP was associated with marital status (p = 0.02) and ANC attendance (p < 0.01). After adjusting for covariates, the use of ≥ 3 doses of IPTp-SP was significantly associated with increased attendance to ANCs (p = < 0.01). Those who attended ≥ 4 ANC visits had six times higher odds of taking ≥ 3 doses of IPTp-SP than those who attended ≤ 3 ANC visits (aOR, 5.91; 95% CI 4.49–7.76; p = < 0.01) (Table 2).

**Table 2:** Association of participant characteristics with optimal use of IPTp-SP

Characteristics	Univariate		Multivariable	
	cOR (95% CI)	p-value	aOR (95% CI)	p-value
Age groups				
18–24	0.65 (0.42–1.01)	0.06	0.96 (0.53–1.74)	0.90
25–29	0.69 (0.43–1.09)	0.11	0.83 (0.48–1.44)	0.50
30–34	0.74 (0.45–1.21)	0.23	0.84 (0.49–1.45)	0.53
> 34	Ref	Ref	Ref	Ref
Marital status				
Married	0.72 (0.55–0.95)	0.02	1.26 (0.91–1.74)	0.16
Unmarried	Ref	Ref	Ref	Ref
Education level				
No formal education	0.40 (0.14–1.15)	0.09	0.41 (0.13–1.32)	0.14
Primary education	1.54 (0.72–3.30)	0.27	1.66 (0.71–3.84)	0.24
Secondary education	1.47 (0.68–3.20)	0.33	1.55 (0.66–3.63)	0.31
Tertiary education	Ref	Ref	Ref	Ref
Attendance to ANC				
≥ 4	5.88 (4.50–7.68)	< 0.01	5.91 (4.49–7.76)	< *0.01*
< 4	Ref	Ref	Ref	Ref
Gravidity				
Primigravida	0.78 (0.59–1.05)	0.10	0.77 (0.49–1.20)	0.24
Secundigravida	0.77 (0.56–1.05)	0.10	0.77 (0.51–1.15)	0.20
Multigravida	Ref	Ref	Ref	Ref

*cOR *crude odds ratio*, aOR* adjusted odds ratio*, ANC* antenatal clinic

### Effect of IPTp-SP doses on placental malaria

Placental malaria by histology was detected in 42 (3.62%) out of the 1161 study participants. Three (7.1%) out of the 42 pregnant women with peripheral malaria had placental malaria (p = 0.002), accounting for 21-fold risk of placental malaria than those without peripheral parasitaemia (aOR, 21.37; 95% CI 4.47–102.08; p < 0.001). The prevalence of placenta malaria with active infection, active-chronic infection and past infection were 2.5%, 0.4% and 0.5% respectively. Out of 397 pregnant women who used ≤ 2 doses of IPTp-SP, 16 (4.0%) of them had placental malaria. For 764 pregnant women who used ≥ 3 doses of IPTp-SP, 26 (3.4%) of them had placental malaria. Sub-optimal doses (≤ 2 doses) of IPTp-SP did not increase the risk of placental malaria among pregnant women (p = 0.97) (Table 3).

**Table 3:** Association between peripheral and placental malaria with IPTp-SP doses

Variable	≤ 2 IPTp-SP doses n = 397	≤3 IPTp-SP doses n = 764	p-value
Peripheral malaria (RDT)			
Positive	2 (0.50)	6 (0.80)	0.72
Negative	395 (99.50)	758 (99.2)	
Placenta malaria by histology			
Infected RBCs	12 (3.0)	19 (2.5)	
Haemozoin	2 (0.5)	4 (0.5)	0.97
Infected RBCs + haemozoin	2 (0.5)	3 (0.4)	
Submicroscopic placental malaria by PCR (n = 286)			
Positive	1 (1.04)	3 (1.58)	1.00
Negative	95 (98.96)	187 (98.42)	
*Plasmodium* species by PCR (n = 4)			
*Plasmodium falciparum*	1 (100.00)	3 (100.00)	N/A
*Plasmodium vivax*	0	0	N/A
*Plasmodium malariae*	0	0	N/A
*Plasmodium ovale*	0	0	N/A

*RDT* malaria rapid diagnostic test*, RBC* red blood cells, *PCR* polymerase chain reaction, *N/A* not applicable

Out of 1119 placental blood samples that were microscopically confirmed to be negative for malaria parasites, 4/286(1.4%) had submicroscopic placental malaria. Of these participants with submicroscopic placental malaria, 3 (1.58%) of them used ≥ 3 IPTp-SP doses while 1 (1.04%) used ≤ 2 IPTp-SP doses. PCR positive samples revealed that, all the submicroscopic infections were due to *P. falciparum* (Table 2).

### Effect of sub-optimal IPTp-SP doses on adverse birth outcomes

Out of 1161 pregnant women, the prevalence of maternal anaemia and fetal anaemia was 43.8% and 10.1%, respectively. Peripheral malaria was significantly associated with maternal anaemia and not fetal anaemia. Women with peripheral malaria had six times risk of maternal anaemia than those who had no malaria infection (aOR, 5.83; 95% CI 1.10–30.92; p = 0.04). In multivariable analysis, the use of ≤ 2 IPTp-SP doses increased the risk of maternal anaemia by 1.36 times higher compared to the use of ≥ 3doses (aOR, 1.36; 95% CI 1.04–1.79; p = 0.02), (Table 4).

**Table 4:** Effect of IPTp-SP doses on maternal anaemia, fetal anaemia and preterm delivery

Outcome		Univariate analysis		Multivariable analysis	
	n (%)	cOR, 95% CI	p-value	aOR, 95% CI	p-value
Maternal anemia					
0–2 doses	187 (47.1)	1.23 (0.96–1.57)	0.10	1.36 (1.04–1.79)	*0.02*^*a*^
≥ 3 doses	321 (42.0)	Ref	Ref	Ref	Ref
Fetal anemia					
0–2 doses	36 (9.1)	0.84 (0.56–1.27)	0.41	0.85 (0.54–1.34)	0.49^b^
≥ 3 doses	81 (10.6)	Ref	Ref	Ref	Ref
Preterm delivery					
0–2 doses	37 (9.3)	1.38 (0.89–2.14)	0.15	1.06 (0.65–1.73)	0.81^c^
≥ 3 doses	53 (6.9)	Ref	Ref	Ref	Ref

^a^ Adjusted for placental malaria, ANC visits, FEFO use, peripheral malaria, mebendazole, ITN, mosquito spray/repellent and gravidity

^b^ Adjusted for placental malaria, ANC visits, FEFO use, peripheral malaria, mebendazole, ITN, mosquito spray/repellent, maternal anaemia, sex of newborn and gravidity

^c^ Adjusted for placental malaria, ANC visits, FEFO use, peripheral malaria, mebendazole, ITN, mosquito spray/repellent, gravidity, maternal anaemia and age of mothers

The use of sub-optimal IPTp-SP doses did not increase the risk of fetal anaemia (cOR, 0.84; 95% CI, 0.56–1.27; p = 0.41). Further analysis revealed that, pregnant women who had anaemia were 2 times at increased risk of delivering anaemic babies (aOR, 1.9; 95% CI 1.31–2.87; p = < 0.01). Other characteristics of pregnant women such as age, marital status, education level, number of ANC visits, use of FEFO, mebendazole, ITN, mosquito spray/repellents, gestation age, gravidity and placental malaria were not associated with maternal and fetal anaemia.

Also, the risk of preterm delivery was not increased by the use of sub-optimal IPTp-SP doses, however, factors such as primigravidity and mosquito spray/repellents were associated with preterm delivery as reported previously [27]. Women who did not use ITN had increased risk of preterm delivery three-times higher than those who used ITN (aOR,3.39; 95% CI 1.078–10.67; p = 0.04). In addition, women who had < 4 ANC visits had two-fold risk of preterm delivery compared to those who attended ≥ 4 ANC visits (aOR, 2.05; 95% CI 1.26–3.33; p = 0.004).

### Effect of sulfadoxine plasma concentration at birth on peripheral and placental malaria and adverse birth outcomes

SDX was detected in 218 (92.0%) out of 237 participants. A majority (60.1%) of those who had detectable SDX used ≥ 3 doses of IPTp-SP. In the analysed samples (n = 237), 79 (33.3%) samples had detectable SDX levels but could not be quantified (plasma concentration was < 3 μg/ml). Three (1.3%) participants had detectable and quantified SDX concentration despite of reporting to have not taken any dose of IPTp-SP throughout their recent pregnancies. Out of 19 (8.0%) women who had undetectable SDX in plasma, 13 (68.4%) used ≤ 2 doses of IPTp-SP. Six (2.5%) participants who reported to have used ≥ 3 doses of IPTp-SP had no detectable SDX in plasma. The overall geometric mean plasma SDX concentration was 10.76 ± 2.51 μg/mL. The geometric mean concentration for women who used ≥ 3 IPTp-SP doses was 10.46 ± 2.50 μg/mL, while those who used ≤ 2 doses was 11.24 ± 2.54 μg/mL. There was no difference in the geometric mean of plasma concentration between women who used sub-optimal versus optimal IPTp-SP doses (p = 0.65) (Fig. 1a).

There was no statistical association between prevalence of peripheral malaria and low SDX concentration (p = 0.37). In addition, the differences in SDX plasma concentration at birth had no effect on placental malaria among the study participants (p = 0.24) (Table 5).

**Table 5:** Effect of plasma Sulfadoxine concentration on maternal malaria and adverse birth outcomes

Variables	Geometric mean, SDX conc. (± SD) μg/mL	p-value
Peripheral malaria		
Positive	6.0 ± 1.28	
Negative	10.84 ± 2.52	0.37
Placental malaria		
Positive	20.02 ± 2.75	0.24
Negative	10.62 ± 2.51	
Maternal Hb, g/dL		
< 11.0	10.19 ± 2.49	0.49
≥ 11.0	11.31 ± 2.54	
Fetal Hb, g/dL		
< 12.5	10.76 ± 2.14	1.00
≥ 12.5	10.76 ± 2.55	
Birth weight (Kg)		
< 2.5	9.90 ± 2.54	0.74
≥ 2.5	10.84 ± 2.52	
Fetal maturity at delivery		
Premature	8.79 ± 2.77	0.29
Mature	11.10 ± 2.47	

*IPTp-SP* intermittent preventive malaria in pregnant women using SDX-pyrimethamine, SDX SDX, *Hb* haemoglobin, *LBW* low birth weight, *NBW* normal birth weight, *SD* standard deviation

**Fig. 2:**
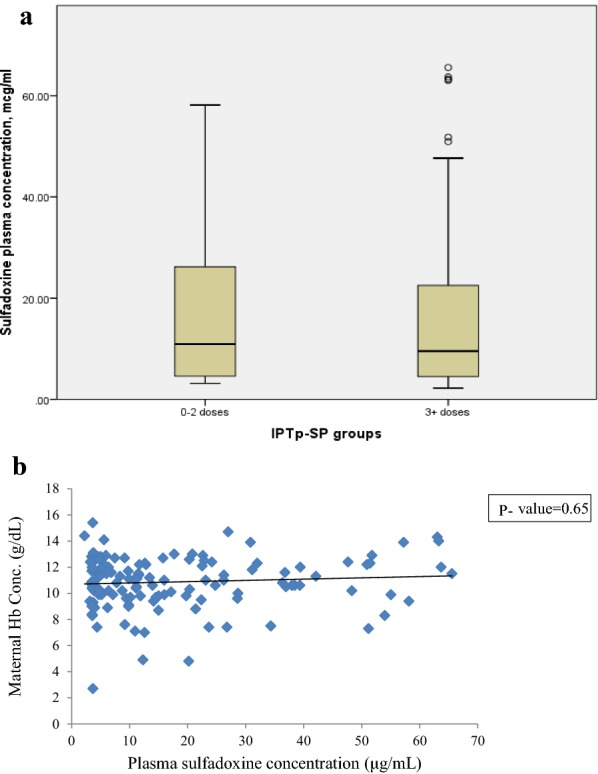
Plasma sulfadoxine concentration and IPTp-SP doses used by pregnant women (**a**); the effect of plasma sulfadoxine concentration on maternal haemoglobin, spearman’s correlation coefficient = 0.02, p = 0.81 (**b**)

There was a slight increase in maternal Hb concentration with plasma concentration of SDX at delivery, but this relationship was weak (spearman’s correlation = 0.02) (Fig. 2b). The geometric mean of plasma SDX between anaemic and non-anaemic women was not statistically different (p = 0.49). Similarly, the differences of geometric SDX concentration at birth was not associated with low birth weight, fetal anaemia and preterm delivery (p > 0.05), (Table 5).

## Discussion

This study highlights the effect of sub-optimal IPTp-SP doses on placental malaria, maternal anaemia and fetal anaemia in malaria hypoendemic region (1.1%). The effect of plasma SP concentration in relation to maternal and birth outcomes was also determined. The prevalence of placental malaria, maternal anemia, fetal anemia and preterm delivery was 3.6%, 43.8%, 10.1% and 7.8% respectively. The use of sub-optimal IPTp-SP doses increased the risk of maternal anaemia by 1.36 times higher than optimal IPTp-SP doses.

Majority of pregnant women reported to have used the recommended optimal doses (≥ 3 doses) of IPTp-SP during pregnancy. The improved uptake of IPTp-SP ≥ 3 could be attributed to the increase in sensitization of SP use for pregnant woman attending the ANC. Previous report on the use of IPTp-SP in Tanzania indicated the low prevalence of 26% for the use of ≥ 3 doses of IPTp-SP which is below 60% target by the ministry responsible for health [28].

This study was conducted in an urban area where the use of mass media in advertising the importance of IPTp-SP use is highly publicized. Moreover, the availability of well-trained health care providers and healthcare facilities being close to households as well as equipped health facilities might have contributed to improved uptake of IPTp-SP in pregnant women. The use of ≥ 3doses of IPTp-SP was significantly associated with ANC attendance. Similar, findings have been reported by other studies indicating that frequent contact with health care providers increased the chances of more intake of SP doses among pregnant women [29].

In this study, there was lower prevalence of placental malaria (3.6%). This is almost half (6.6%) of the prevalence of placental malaria that was reported in the same catchment area in 2010/2012 [30]. The reduced prevalence of malaria in pregnant women can be explained by increased uptake of IPTp-SP and substantial reduction of overall malaria prevalence in this area. The optimal use of IPTp-SP contributes to about 50% reduction in placental malaria [1]. Malaria transmission in this area had declined over time which could have resulted in the reduced prevalence of placental malaria by more than 50% [2, 3, 30].

The current study observed an increased risk of placental malaria among pregnant women with peripheral malaria. The risk of placental malaria among pregnant women with peripheral malaria has been well studied [23, 31–33]. The peripheral malaria infections were asymptomatic which could progress to severe malaria if diagnosis was delayed. Despite the reduced malaria transmission, maternal malaria infections count a significant risk of placental malaria which consequently results in detrimental birth outcomes. Therefore, the lack of statistical difference between those who received optimal and sub-optimal doses for IPTp-SP may not be translated clinically. Similarly, the SDX plasma concentration was not associated with any risk of MiP. The lack of effect of SDX on MiP could be attributed to high level of resistance [34].

SDX was used as a proxy of uptake of SP owing to its long half-life of about 10 days [7, 8]. SDX is found in high concentration in plasma than RBCs and can be detected at delivery for IPTp-SP doses taken from 20 weeks of gestation age. Therefore, detection of SDX in plasma was used to authenticate the information for the use of IPTp-SP by pregnant women that was recorded in the ANC cards. The study noted false positives and negative in SP data. This observation could indicate possibility of incorrect documentation for the uptake of IPTp-SP in the ANCs. The providers guide on antenatal care in Tanzania mainland recommends proper documentation of IPTp-SP doses after administration under DOT [35]. However, in case of stock-outs of SP, pregnant women tend to purchase SP from private community pharmacies, and this may go undocumented [29]. Indeed documented uptake of IPTp-SP is in the ANCs is reported to be more reliable than self-reporting by pregnant women [36]. Detection of SDX at delivery predicts the exposure of pregnant women to SP that was used especially within the wash out period of the drug. However, lack of effect of plasma SDX concentration on placental malaria could be attributed to few malaria cases among the study participants.

The findings of this study, indicate that, the use of sub-optimal doses of IPTp-SP increased the risk of maternal anaemia in low transmission settings. The association of sub-optimal doses of SP with anaemia may result because these women received fewer FEFO doses or if they attended fewer ANC visits. Also, if a women delivered preterm she may not have been able to receive a third/fourth IPTp-SP dose. As Hb is often lower in mid-pregnancy due to plasma volume dilution, this might also relate to the observation of lower Hb in women received ≤ 2 doses. These results are in contrast to findings by Mosha and his colleagues who reported lack of effect of IPTp-SP doses on maternal anaemia [37]. The difference in the findings between the two studies could be explained by differences in the study design and sample size. In the study by Mosha et al., only 89 pregnant women were studied compared to 1161 in the present study. SP is an antifolate that exhibits antibacterial effect. This has added advantage for SP when used for IPTp despite of its compromised effectiveness against *P. falciparum* [38, 39]. Several studies have reported the role of bacteraemia in causing anaemia [40, 41]. Infections alter the ferrokinetics resulting into reduced iron binding capacity and increases iron clearance up to three times (reduced plasma iron level) [42].

Individuals who are anaemic are more vulnerable to infections [41]. In this study, 43.8% of pregnant women were anaemic and, therefore, susceptible to infections. Fifteen (1.3%) pregnant women had fever without malaria infection, indicating presence of non-malaria infections in among study participants. SP when taken at therapeutic doses (and optimal doses) could have cleared none detected and untreated bacterial infections. Therefore, it is probable that, pregnant women who used sub-optimal doses were at high risk of bacterial infections which could not be cleared by sub-optimal IPTp-SP doses. This could enumerate the added advantage of IPTp-SP regardless of reduced malaria transmission in developing countries, where parasitic and bacterial infections are predominant and may occur concurrently [13]. Further, longitudinal studies should be designed to demonstrate the effect of sub-optimal versus optimal IPTp-SP doses on maternal vaginal microbiota and maternal birth outcomes thereof.

In this study, pregnant women who were anaemic had increased risk of delivering anaemic babies (fetal anaemia). This association has also been reported in previous studies where the risk of fetal anaemia increased with severity of maternal anaemia [43, 44]. Fetal iron stores and active transport system across the placenta is responsible for protecting the fetus from iron deficiency. Studies have reported low cord blood ferritin level among infants born to mothers with low ferritin levels [45]. The risk of fetal anaemia is also increased by malaria and HIV infections [46, 47]. The study excluded HIV infected pregnant women, and malaria cases were very few. However, these few malaria cases could have significant adverse consequences in the study area with low malaria prevalence.

The use of sub-optimal or optimal doses of IPTp-SP was not associated with increased risk of fetal anaemia in this study. These findings are similar to those reported by Abrams et al. [44], indicating the lack of association between the use of IPTp-SP and risk of fetal anaemia. However, the results of this study are in contrast with the study by Harrington et al, who demonstrated increased risk of fetal anaemia with the use of IPTp-SP [47]. The later study was conducted in Muheza, Northern Tanzania which has high prevalence of malaria parasites harboring sextuple mutants haplotypes (*Pfdhps*-A581G in combination with the quintuple mutant) [16]. About 90% of plasma SDX can be found in cord plasma; however, this does not seem to inhibit the in utero erythropoietic process in fetus [48]. Despite of that, SP accounts to only 3% of anaemia cases and can cause megaloblastic anaemia if given at high doses in the general population [48]. In the current study, SP for IPTp was given at therapeutic doses as recommended by the WHO [5].

Since 90.7% of women who used at least one dose of IPTp-SP had detectable SDX at the time of delivery, this observation increased our confidence in using the documented number of doses on ANC cards and that majority of them took the last dose by DOT at least at the 28th week of gestation. Presence of SDX in plasma during pregnancy (from early in the 2nd trimester) is necessary to clear peripheral and placental malaria that subsequently reduces the risk of maternal anaemia and adverse birth outcomes [44, 49].

## Limitation

This was a cross sectional study that involved large number of pregnant women at delivery. Adherence on the use of IPTp-SP could not be assessed as the number of doses reported was based on SP doses documented in the ANC cards. Despite this limitation, the detection of plasma SDX at delivery was useful in authenticating the information on the use of IPTp-SP among study participants. Due to few women with positive submicroscopic malaria parasites, the impact of IPTp-SP doses on submicroscopic placental malaria was not ascertained. Assessment of plasma SDX was done at delivery. Plasma SDX samples taken at different time intervals during pregnancy would have provided more reliable results to also assess adherence to IPTp-SP among pregnant women. However, based on the long half-life of SDX, the results of the present study are relevant for IPTp-SP doses that were taken from 20th to 40th week of gestation age.

## Conclusion

The use of sub-optimal doses of IPTp-SP increased the risk of maternal anaemia in malaria hypoendemic region in Tanzania. The use of sub-optimal IPTp-SP doses was not associated with increased risk of malaria parasitaemia, fetal anaemia and preterm delivery among pregnant women in low malaria transmission setting. Uptake of optimal doses (≥ 3 doses) of IPTp-SP and complementary interventions should continue while searching for alternative malaria preventive strategies to IPTp-SP in malaria hypoendemic regions. Early malaria case detection and prompt treatment with effective anti-malarial drugs is highly encouraged to prevent severe malaria and deleterious clinical outcomes in pregnant women.

## Data Availability

The dataset generated and/or analysed during this study is available from the corresponding author upon reasonable request.

## Abbreviations

IPTp: Intermittent preventive treatment of malaria in pregnant women
MiP: Malaria in pregnancy
SP: Sulfadoxine-pyrimethamine
SDX: Sulfadoxine
HPLC: High performance liquid chromatograph
PCR: Polymerase chain reaction
